# Current and Future Applications of Computational Fluid Dynamics in Coronary Artery Disease

**DOI:** 10.31083/j.rcm2311377

**Published:** 2022-11-04

**Authors:** Alessandro Candreva, Giuseppe De Nisco, Maurizio Lodi Rizzini, Fabrizio D’Ascenzo, Gaetano Maria De Ferrari, Diego Gallo, Umberto Morbiducci, Claudio Chiastra

**Affiliations:** ^1^PoliTo^BIO^Med Lab, Department of Mechanical and Aerospace Engineering, Politecnico di Torino, 10129 Torino, Italy; ^2^Department of Cardiology, Zurich University Hospital, 8091 Zurich, Switzerland; ^3^Department of Medical Sciences, Division of Cardiology, AOU Città Della Salute e Della Scienza, University of Turin, 10124 Turin, Italy

**Keywords:** coronary artery disease, atherosclerosis, computer model, computer simulation, computational hemodynamics, virtual FFR, wall shear stress, helicity

## Abstract

Hemodynamics interacts with the cellular components of human vessels, influencing function and healthy status. Locally acting hemodynamic forces have been associated—by a steadily increasing amount of scientific evidence—with nucleation and evolution of atherosclerotic plaques in several vascular regions, resulting in the formulation of the ‘hemodynamic risk hypothesis’ of the atherogenesis. At the level of coronary arteries, however, the complexity of both anatomy and physiology made the study of this vascular region particularly difficult for researchers. Developments in computational fluid dynamics (CFD) have recently allowed an accurate modelling of the intracoronary hemodynamics, thus offering physicians a unique tool for the investigation of this crucial human system by means of advanced mathematical simulations. The present review of CFD applications in coronary artery disease was set to concisely offer the medical reader the theoretical foundations of quantitative intravascular hemodynamics—reasoned schematically in the text in its basic (i.e., pressure and velocity) and derived quantities (e.g., fractional flow reserve, wall shear stress and helicity)—along with its current implications in clinical research. Moreover, attention was paid in classifying computational modelling derived from invasive and non-invasive imaging modalities with unbiased remarks on the advantages and limitations of each procedure. Finally, an extensive description—aided by explanatory figures and cross references to recent clinical findings—was presented on the role of near-wall hemodynamics, in terms of shear stress, and of intravascular flow complexity, in terms of helical flow.

## 1. Introduction

Following nucleation, coronary atherosclerotic plaques differentiate into several clinical phenotypes. Whilst most of the plaques will remain uneventful lifelong, a proportion of them will progress into flow-limiting lesions or become unstable, rupture and provoke acute coronary syndromes [1, 2]. For its epidemiological impact, the understanding of the mechanisms underlying coronary atherosclerotic plaque onset, progression and rupture is of clinical significance.

Although extensive scientific efforts, prediction of plaque formation, evolution and vulnerability remains equivocal. Firstly, despite the arguably systemic distribution of vascular inflammation and the systemic effect of cardiovascular risk factors, plaque nucleation appears to be a local phenomenon. In fact, atherosclerotic plaques cluster in preferential anatomic regions (e.g., coronary, carotid or lower-limb arteries) and at preferential vascular sites (e.g., inner curvatures, bifurcations and T-junctions) [3, 4]. Secondly, several studies linked plaque composition and inflammatory plaque infiltration footprints with a vulnerable phenotype (see e.g., [2, 5, 6]). However, the registered elevated senescence rate of those lesions identified as vulnerable have failed so far to justify pre-emptive therapeutic interventions aiming at stabilizing the plaque with an improvement of patient long term outcome [6]. Thirdly, increased transcoronary pressure gradients were associated not only with myocardial flow impairment [7] but also with plaque destabilization [8], thus suggesting a harmful role of trans-stenotic forces acting across flow-impairing plaques [3]. Lastly, coronary intervention targeting myocardial perfusion deficits failed to reduce occurrence of major adverse cardiac events compared to optimal medical treatment [9], indicating the prevention of acute coronary events rather than the sole treatment of myocardial ischemia as more relevant target therapy to impact patient outcome.

Coronary atherosclerotic plaques experience complex biomechanical forces during each cardiac cycle as the result of the interaction between the pulsatile blood flow with the moving artery geometry [3]. The role of local blood flow-vessel interaction has gained scientific momentum becoming subject to extensive investigation, especially in relation with vessel remodeling and atherosclerotic plaque evolution in the coronary vascular bed. Given the impossibility of a direct *in vivo* measurement of those flow-related quantities acting as local biomechanical stimuli at the blood-endothelium interface, increasingly refined and personalized computer models able to realistically capture cardiovascular flows have been developed [10] and applied to study intracoronary hemodynamics [11]. Consequently, hemodynamic factors influencing vascular homeostasis as well as atherosclerotic lesion development have been proposed, hence providing evidence to the so-called ‘hemodynamic risk hypothesis’ of atherosclerosis [3, 12]. According to this hypothesis, local onset and progression of atherosclerosis can be promoted by local blood flow disturbances.

However, the integration of computer model-based intracoronary hemodynamic data within the clinical practice is mainly hampered by a demanding computational cost to run simulations, especially when compared to current diagnostic imaging acquisitions. This has prevented the use of computational hemodynamics in large clinical studies, which in turn would be required to prove the utility of computer based hemodynamic modelling, setting up a vicious cycle. Moreover, computer based hemodynamic modelling is perceived by cardiologists as a technology for which most of them have never been trained and this represent a barrier to its adoption.

## 2. Aims and Structure of the Present Writing

The present review of the literature aims to broaden the understanding of computer based hemodynamic modelling and to highlight the opportunities opened by its clinical application in cardiology. More specifically, it offers the non-technical, medical reader (i) a simplified but rigorous explanation of coronary artery hemodynamics, (ii) a broad overview on the applications of computational fluid dynamics (CFD) based modelling to coronary artery hemodynamics, and (iii) the current level of scientific evidence and of implementation of CFD in the clinical practice.

After a brief overview on the complexity of coronary artery hemodynamics in Section 3, the principles of CFD application to the human coronary system and the generation of flow simulations are presented in a step-by-step fashion in Section 4. From here, a detailed description of CFD applications concerning the assessment of intracoronary pressure is presented in Section 5, with distinction between methods based on invasive and non-invasive imaging modalities. In this part of the manuscript, ample space is dedicated to the discussion of various existing CFD based tools and their clinical role. Near-wall and intravascular flow patterns will be the main focus of Section 6, where preclinical and early clinical applications will be presented. Finally, limitations of the CFD-based current methodology and future perspectives (including artificial intelligence) will be discussed in Section 7.

## 3. Features of Intracoronary Hemodynamics

Coronary artery hemodynamics can be seen as a system characterized by a remarkable level of complexity. A main source of complexity is the anatomy of the coronary tree, which presents a pronounced tapering (especially in the left coronary vasculature) and follows an asymmetrical fractal dichotomizing pattern [13], where flow distribution at bifurcations is not equal among the two daughter ramifications, namely distal main vessel and side branch [14]. Acting as a flow divider, the presence of the coronary bifurcation carina literally splits the incoming flow rate into two asymmetric flows with velocity profiles modelled by the local geometry [15]. In this region, the sudden changes in velocity direction and magnitude of the flowing blood lead to complex patterns usually characterized by flow separation and reattachment, with direct effect on endothelial cell distribution, shape and function [16] as well as on circulating cell prolonging their adhesion time to the endothelium [17]. In addition, variability in the distribution of diagonal and marginal branches is commonly observed. Tortuous and ectatic vascular segments are frequently encountered [18]. Another source of complexity is represented by the dynamic vasomotion autoregulation characterizing both epicardial coronary arteries and smaller arteriolae (i.e., with a cross-sectional diameter <400 μm). In fact, vascular smooth muscle cell contraction is finely tuned by circulating and endothelial-derived vasoactive substances (e.g., nitric oxide and adenosine diphosphate) released in case of changes in metabolic demands or perfusion [3]. A further element of complexity is represented by the myocardial mechanics, where the systolic myocardial contraction interacts on coronary vessels causing (i) pulsatile and complex flow patterns with a prominent diastolic component [10], (ii) a cyclic longitudinal vessel shrinkage (which adds on the natural tortuosity of the epicardial vessels), and (iii) a cyclic transversal compression of the intramural segments of epicardial arteries [19]. Finally, coronary driving pressure strictly depends on the systemic filling status and the cardiac function [20].

Hence, capturing the complexity of coronary artery physiology into a virtual environment for blood flow simulation represents for sure a singular challenge.

## 4. Basics of Computational Fluid Dynamics

Initially developed in the middle of the last century to solve complex engineering problems through the execution of numerical simulations, CFD solves numerically in space and time the physics equations governing fluid motion, thus allowing to mathematically describe and analyze flow fields also in complex geometries [21]. To be clearer, the nature of the governing equations describing the time-varying motion of fluids, namely the Navier-Stokes equations (expressing the conservation laws of fluid dynamics), prevents their analytical resolution in case of complex 3D fluid domains. Thus, numerical schemes, typically based on the finite volume or finite element method, are adopted to solve the equations in their discretized form [21]. Applied with high spatial and temporal resolution to the simulation of blood flow patterns, the combination of CFD with clinical imaging represents for cardiologists a powerful technology to quantitatively assess hemodynamic forces acting locally on the endothelium.

**Fig. 1.**
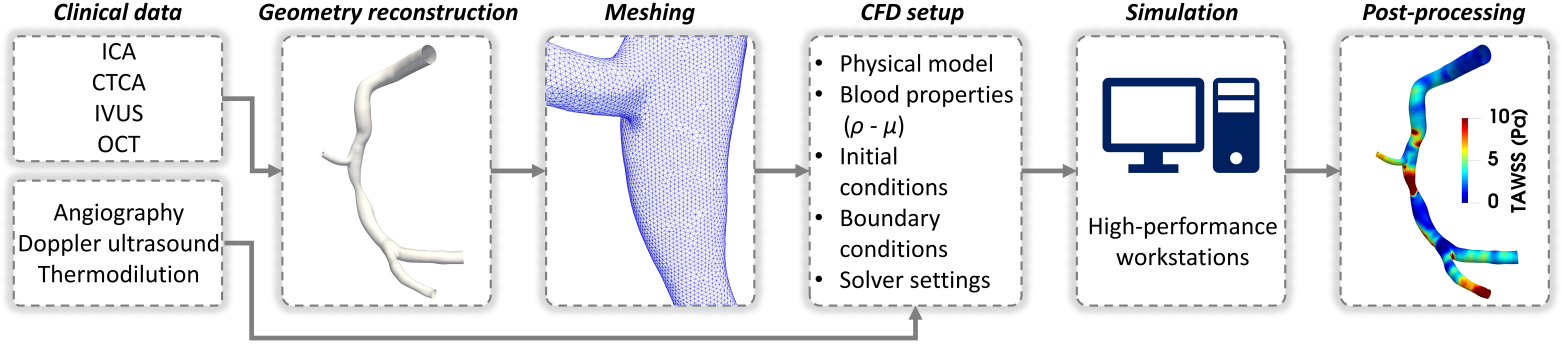
**Workflow of patient-specific computational fluid dynamics simulations for an explanatory case of diseased right coronary artery**. The artery model belongs to a patient recruited during the clinical trial RELATE (ClinicalTrials.gov Identifier: NCT04048005). ICA, invasive coronary angiography; CTCA, computed tomography coronary angiography; IVUS, intravascular ultrasound; OCT, optical coherence tomography; ρ, blood density; μ, blood dynamic viscosity; TAWSS, time-average wall shear stress.

To obtain robust results, CFD tools require several steps to be appropriately executed, including vascular geometry reconstruction, boundary conditions (BCs) definition, and material properties setting; all these steps concur to determine the reliability of the simulation results [22, 23]. Fig. 1 summarizes the main steps of patient-specific CFD simulations for the analysis of the coronary artery hemodynamics.

Firstly, the patient-specific 3D coronary artery geometry is reconstructed from conventional invasive coronary angiography (ICA), computed tomography coronary angiography (CTCA), or from the fusion of one of the previous imaging modalities with intravascular imaging techniques (i.e., intravascular ultrasound – IVUS or optical coherence tomography – OCT). Clinical imaging is used to obtain information about the vascular segments of interest with resolutions close to 1 mm or even lower, which is of considerable importance for the accurate characterization of local coronary hemodynamics [23]. This information will be used to create the CFD model, defining the fluid domain of interest (Fig. 1).

Secondly, the so-obtained 3D fluid domain of interest is subdivided into smaller sub-domains called ‘elements’ (i.e., outputs of the discretization process, also known as meshing process), where the equations of fluid motion are solved in their discrete form. The discretization of the Navier-Stokes equations is necessary since their resolution in complex 3D fluid domains cannot be analytically obtained. By that, a system of non-linear partial differential equations is transformed into a system of algebraic equations that can be solved numerically. Finer grid spacing (i.e., smaller element size) is usually required for complex vascular regions, where larger variation in velocity and/or pressure profiles are expected. On the contrary, larger element size might be used in vascular regions where low spatial variability of the hemodynamic quantities is expected. High spatial resolutions imply computationally expensive simulations, usually requiring the adoption of high-performance computing technology.

**Fig. 2.**
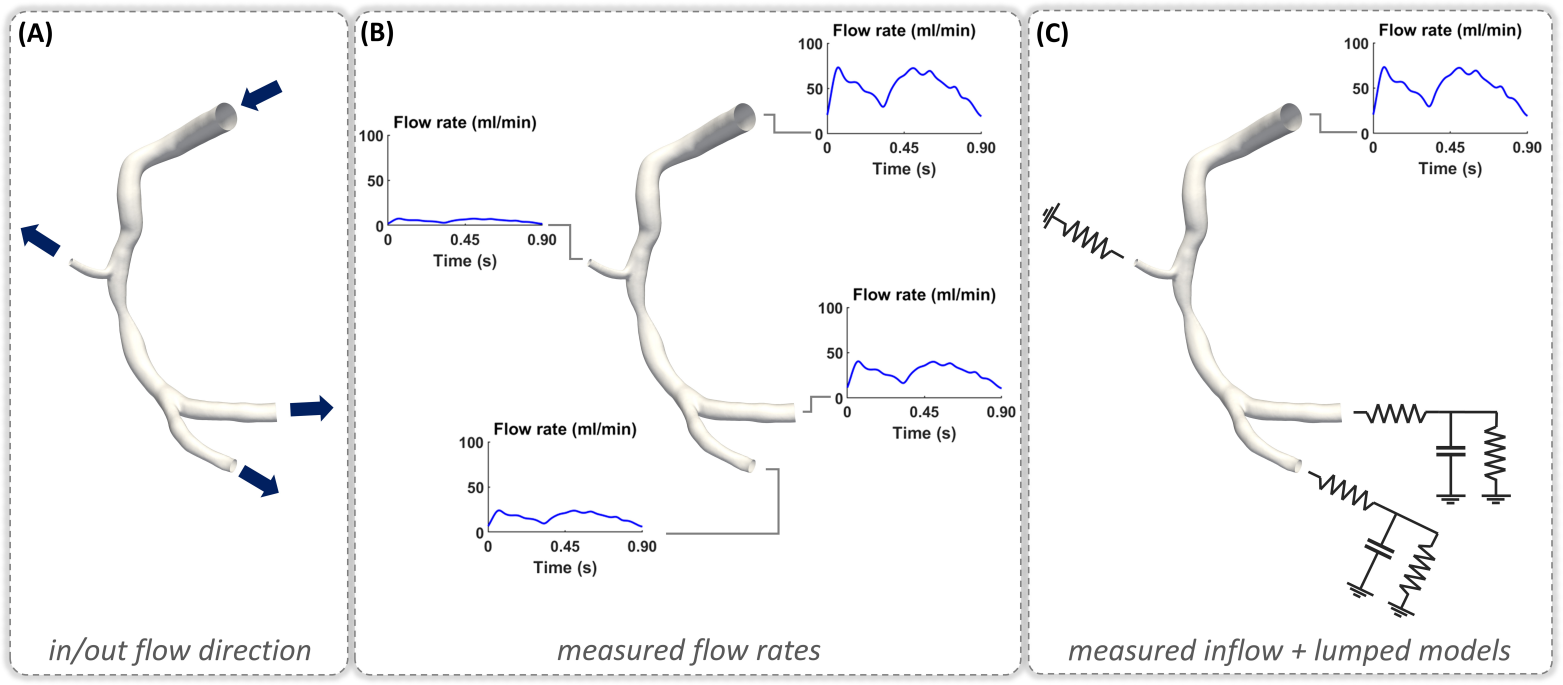
**Explanatory strategies of computational fluid dynamics (CFD) boundary conditions (BCs) that can be prescribed to a diseased right coronary artery model**. (A) In/out flow direction panel: the dark blue arrows display the direction of blood flow at each inlet/outlet boundary cross-section of the vessel model. (B) Measured flow rates panel: blood flow rate waveforms extracted from imaging or *in vivo* measurement techniques are prescribed at each model inlet/outlet cross-section of the vessel model. The measured blood flow rates applied as BCs are shown. (C) Measured inflow + lumped models panel: BCs are defined by coupling measured clinical data, available at the inflow section, with lumped parameter circuit models describing the peripheral vascular resistance and compliance. The diseased right coronary artery belongs to a patient recruited during the RELATE clinical trial (ClinicalTrials.gov Identifier: NCT04048005).

Thirdly, the CFD simulation is set up by defining *a priori* the physical model, the blood material properties (in terms of blood density and viscosit*y*), the initial conditions and BCs contextualizing as much as possible the physical phenomenon, and the solver numerical settings. CFD simulations can be carried out under constant (steady-state) or pulsatile (unsteady-state) flow conditions, depending on the quantities we are interested in (e.g., pressure rather than shear stress profiles). Blood is assumed as homogeneous, incompressible fluid with constant density. In most cases, blood viscosity is described through non-Newtonian rheological models able to replicate its shear-tinning behavior (e.g., Carreau or Quemada models) [24]. The use of the Newtonian model is also accepted, since it proved to be likely appropriate for hemodynamics simulation in arterial domains characterized by high shear rates (>50 $s^{-1}$) and low particle residence time [25]. A proper description of the hemodynamic conditions at the inlet/outlet boundaries of the model, in terms of prescribed values of velocity or pressure, is required for the resolution of the governing equations of fluids (Fig. 2). Inlet/outlet BCs of the coronary artery model can be extracted using subject-specific clinical data. In detail, velocity and/or flow rate data are usually obtained from clinical imaging techniques, such as angiography-based thrombolysis in myocardial infarction (TIMI) frame count [26], as well as *in vivo* measurement techniques, such as intracoronary Doppler ultrasound [27] and intracoronary continuous thermodilution [28]. Subject-specific pressure data can be derived from *in vivo* pressure wire measurements [29]. If such data are not available, generic flow/pressure references from literature can be prescribed. The latter make the CFD model weakly tailored to the specific subject, but that not necessarily implies less affordable simulation results (it depends on the simulated quantities of interest). An alternative strategy to define BCs consists in the coupling of the vessel inlet and outlets to lumped parameter circuit models (e.g., the Windkessel model), which mimics aortic driving forces and peripheral resistances and compliances, respectively (Fig. 2) [30]. The coronary artery wall can be considered as a deformable structure by the simulation of vessel compliance and myocardial contraction-induced vessel deformation during the cardiac cycle (e.g., [31]), or as rigid structure (e.g., [32, 33]). Usually, the latter option is adopted, as it has been demonstrated that cycle-average hemodynamic quantities are less impacted by vessel compliance and deformation [34, 35].

Fourthly, once properly set, the CFD simulation is run. The discretized governing equations of fluid motion are iteratively solved to reach a solution for which residual errors in velocity and pressure fall below a certain threshold (pre-selected by the user based on the accuracy that is considered adequate for numerically solving the equations).

At last, simulation results are post-processed to extract the hemodynamic quantities and indexes of interest. Both intracoronary pressures and flows can be quantified, describing their behavior within the streaming medium and along the blood-vessel interface (i.e., near-wall hemodynamic quantities). Accordingly, in the following sections clinical applications of CFD will be addressed separately for computational simulation of coronary pressure (Section 5) and coronary flow patterns (Section 6). Additionally, the main limitations of CFD application in coronary arteries will be discussed in Section 7.

## 5. CFD Based Intracoronary Pressure Distribution Evaluation

Plaque infiltration and the resulting inward vascular remodeling (according to Glagov’s hypothesis [36]) impact vessel conductance and generate intravascular pressure gradients [3, 37]. In turn, increased vascular resistance impairs coronary flow downstream of the stenosis. This can be quantitatively assessed through invasive measurement in terms of fractional flow reserve (FFR) as ratio between hyperemic distal coronary and aortic pressures: a flow impairment higher than 20% during hyperemia—which translated in a FFR value lower than 0.80—was associated with myocardial ischemia and with adverse clinical outcomes, thus justifying coronary interventions aiming at resolving flow-impairing coronary lesions [8, 38]. The (assumed) linear relationship between coronary flow and pressure under hyperemia condition was empirically verified by the evidence of constant microvascular resistance during maximal pharmacological hyperemia, thus allowing the measurement of epicardial pressure gradients without the interference of variations in microvascular pressure [39, 40]. More recently, non-hyperemic pressure ratio (NHPR) indices have been developed and successfully validated against FFR [41]. Similarly to FFR, NHPRs measure the status of epicardial vessel conductance, but without the need of administration of hyperemic agents. This is possible given the phasic behavior of microvascular resistances along the cardiac cycle and their stabilization in specific phases of the diastole [42, 43].

Both FFR and NHPRs represent valid solutions to assess coronary perfusion in relationship to the status of epicardial impedance and are highly recommended from international guidelines for the functional assessment of intermediate-grade coronary lesions (typically around 40–90% stenosis) [44]. However, given their invasiveness and the perceived additional procedural time and costs, clinical uptake of intracoronary pressure measurement remains low (<15%) [45] and highly variable among healthcare systems [46]. To overcome the limitations hampering the diffusion of intravascular measurements, alterative solutions exploring derivation of intracoronary pressure profiling in a pressure wire-free manner (e.g., from non-invasive imaging modalities or from the integration of coronary imaging with CFD) have been proposed.

### 5.1 Intracoronary Pressure Evaluation Based on Invasive Coronary Angiography

**Fig. 3.**
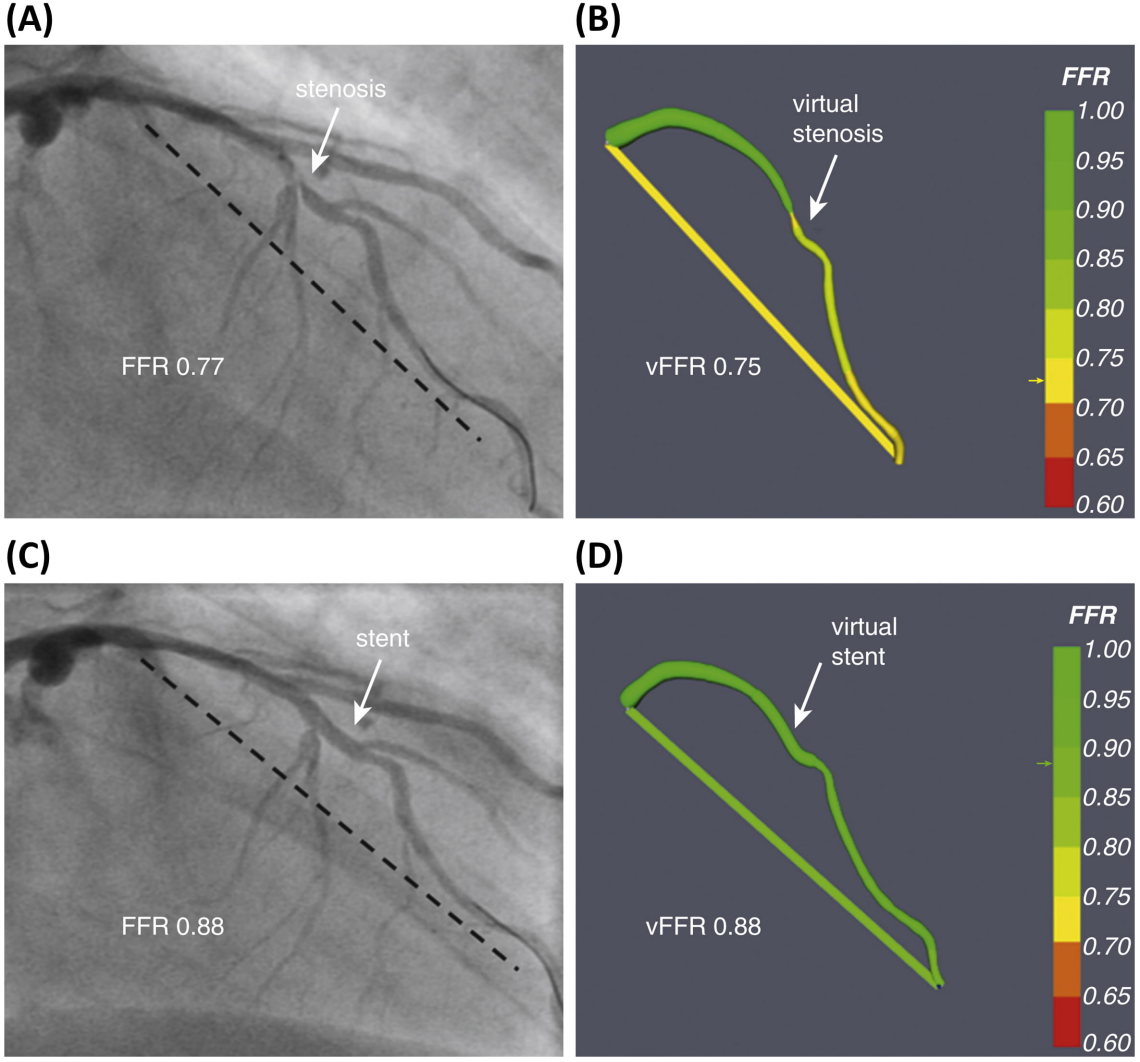
**Explanatory case showing the typical output obtained through the ${\mathrm{VIRTUheart}}^{\text{𝐓𝐌}}$ system for the computation of the vFFR**. (A) A 66-year-old man presented with chronic stable angina. The left anterior descending (LAD) coronary artery had a severe mid vessel stenosis (arrow). The measured FFR between the proximal and distal points (dashed line) was 0.77. (B) Angiograms were used to model the vFFR by using the ${\mathrm{VIRTUheart}}^{\text{TM}}$ system, which was calculated to be 0.75 over the same vessel segment. This is displayed in false color yellow, the straight yellow line connecting the same 2 points between which the vFFR was calculated, exactly matching the 2 spots marked by the dashed line in (A). (C) After implantation of a 2.75 × 18 mm stent at the stenosis, the measured FFR was 0.88 over the same segment. (D) Virtual coronary intervention using the VIRTUheart system was then used to implant a virtual 2.75 ×18 mm stent, and the recalculated vFFR was 0.88, corresponding to the green line connecting the 2 points. Reprinted with permission from Gosling RC, Morris PD, Silva Soto DA, Lawford PV, Hose DR, Gunn JP. Virtual Coronary Intervention: A Treatment Planning Tool Based Upon the Angiogram. JACC Cardiovasc Imaging. 2019; 12(5): 865–872. doi: 10.1016/j.jcmg.2018.01.019 [49] (http://creativecommons.org/licenses/by/4.0/).

3D vessel reconstructions based on two or more orthogonal coronary angiograms were implemented to compute the so-called ‘virtual’ FFR (vFFR) [47]. Pioneering the field, Morris and colleagues developed and validated an effective CFD solution for angiography-based vFFR called ${\mathrm{VIRTUheart}}^{\text{TM}}$ [48]. This CFD solution follows the general workflow summarized in Fig. 1. More in detail, firstly a 3D geometry of the diseased coronary artery is reconstructed from two angiograms as close to 90 degrees apart. Secondly, the vessel geometry is discretized and the CFD model is set up within the commercial software CFX (Ansys Inc, Canonsburg, PA, USA) by applying generic BCs. In this regard, a population-based, generalized pulsatile pressure waveform is prescribed at the inlet. Windkessel models with values of resistances and compliance averaged over the available patients’ data is applied at the outlets. Lastly, CFD simulations are run and the vFFR is quantified. The ${\mathrm{VIRTUheart}}^{\text{TM}}$ CFD solver reproduced physiological lesion significance with excellent accuracy (>90%) [48, 49]. However, the transient CFD simulations of this tool resulted in long processing time (>24 hours). Hence, ‘faster’ solutions based on steady-state CFD simulations for the identification of the parameters of simplified fluid dynamics mathematical models (i.e., lumped parameter models) were developed with significant reduction of computational time (<4 min) [50]. Furthermore, a recent update to the software allowed the virtual simulation of stenting and accurate post-stenting FFR prediction (Fig. 3) [49]. The software currently remains for research use only [51].

Differently from the time-consuming CFD approach, several methods rely on a simplification of the governing equations of fluid motion to describe the hemodynamic features within the coronary artery. The analytical solution of those simpler equations (e.g., Bernoulli’s and Poiseuille’s equations) may provide the needed hemodynamic quantities for fast vFFR computation. Three software solutions based on these methods are currently commercially available in Europe, namely the Cardiovascular Angiographic Analysis System for Vessel CAAS-vFFR (Pie Medical, Maastricht, The Netherlands), the quantitative flow reserve QFR (Medis Medical Imaging, Leiden, The Netherlands and Pulse Medical Technology Inc., Shanghai, China) and the FFRangio (CathWorks Ltd., Kfar-saba, Israel) [52, 53, 54]. In addition to the CE mark, QFR has also received the approval by the US Food and Drug Administration (FDA). Table 1 (Ref. [52, 53, 54, 55, 56, 57, 58, 59, 60, 61, 62, 63, 64, 65, 66]) summaries available clinical evidence for the software mentioned above.

**Table 1.** **Commercially available software for computation of fractional flow reserve and their main clinical studies**.

CFD Solver	Model domain and mathematical solution	Study	Type of Study	Year	Sample size and population studied	Primary endpoint
CAAS-vFFR, Pie Medical	Angiography-based 3D-QCA. Analytical equation accounting for viscous and flow separation pressure losses, with empirically determined coefficients. The inlet coronary velocity, derived from patient-specific aortic rest pressure and 3D vessel geometry, is assumed to be preserved along the vessel segment.	FAST I Study [53]	Retrospective, two-centre	2020	100 vessels with intermediate lesions (30–70%DS) from 100 patients with CCS or NSTE-ACS	Agreement against invasive FFR (FFR cut-off ≤ 0.80):
						r = 0.89, *p *< 0.001
						BA mean difference: 0.01 ± 0.036
						AUC 0.93 [95% CI: 0.88–0.97], *p *< 0.001
		FAST Extend [55]	Retrospective, two-centre	2021	same as FAST I, N = 912	r = 0.89
						AUC 0.94 [95% CI: 0.92–0.97]
		FAST II Study [56]	Prospective, multicentric	2021	same as FAST I, N = 334	r = 0.74, *p *< 0.001
						BA mean difference: 0.003 ± 0.064
						AUC 0.93 [95% CI: 0.90–0.96], *p *< 0.001
		FAST III Study (NCT04931771)/on-going	Non-inferiority RCT (35 European sites)	2021–2024	2228 vessels with intermediate lesions (30–80%DS) in pts with CCS randomized 1:1 towards FFR or vFFR-guided revascularization	Composite of all-cause death, any myocardial infarction, or any revascularization at 1 year post-randomization.
		LIPSIA-STRATEGY (NCT03497637)/on-going	Non-inferiority RCT (7 German sites)	2020–2021	1926 vessels with intermediate lesions (40–80%DS) in pts with stable angina or ACS randomized 1:1 towards FFR or vFFR-guided revascularization	Composite of cardiac death, non-fatal myocardial infarction, or any unplanned revascularization at 1 year post-randomization.
QFR, Medis Medical Imaging Pulse Medical Imaging	Angiography-based 3D-QCA. Analytical equations based on a quadratic relationship between pressure drop and hyperaemic flow velocity. Similar coronary flow velocity at inlet and outlet BCs. Absence of pressure losses along normal coronary segments. Empiric hyperaemic flow velocity of 0.35 m/s (fQFR). TIMI frame counting-derived contrast velocity at baseline (cQFR) and under hyperaemia (aQFR).	FAVOR Pilot Study [57]	Prospective, multicentric	2016	84 vessels with intermediate lesions (30–80%DS) from 73 patients with CCS	Agreement cQFR against invasive FFR (FFR cut-off ≤0.80):
						r = 0.77, *p *< 0.001
						BA mean difference: 0.001 ± 0.059, *p* = 0.9
						AUC 0.92 [95% CI: 0.85–0.97]
		Stähli *et al*. [58]	Retrospective, single-centre	2018	516 vessels with intermediate lesions (40–70%DS) from 436 patients with CCS	r = 0.82, *p *< 0.001
						BA mean difference: 0.01 ± 0.06
						AUC 0.86 [95% CI: 0.83–0.89]
		FAVOR II Europe-Japan [59]	Prospective, multicentric	2018	317 vessels with intermediate lesions (30–80%DS) from 329 patients with CCS	r = 0.83, *p *< 0.001
						BA mean difference: 0.01 ± 0.06
						AUC 0.92 [95% CI: 0.89–0.96], *p *< 0.001
		FAVOR III China [60]	Superiority RCT (26 Chinese sites)	2021	3825 vessels with intermediate lesions (50–90%DS) in pts with CCS or ACS randomized 1:1 towards angiography- or QFR-guided revascularization	Composite of death from any cause, myocardial infarction, or ischaemia-driven revascularisation at 1 year:
						QFR-guided group: 5.8%
						Angiography-guided group: 8.8%
						HR 0.65 [95% CI: 0.51–0.83], *p* = 0.0004, driven by fewer myocardial infarctions and ischaemia-driven revascularisations
		FAVOR III Europe-Japan (NCT03729739)/on-going	Non-inferiority RCT (40 international sites)	2018–2023	2000 vessels with intermediate lesions (30–80%DS) from patients with CCS randomized 1:1 towards FFR- or QFR-guided revascularization	Composite of death from any cause, any myocardial infarction, or any unplanned revascularization at 1 year post-randomization.
${\mathrm{FFR}}_{\text{angio}}$, CathWorks Ltd.	Angiography-based 3D coronary tree reconstruction (including bifurcations). Analytical equation based on Poiseuille’s law.	Pellicano *et al*. [54]	Prospective, multicentric	2017	203 vessels with intermediate lesions (50–90%DS) from 184 patients with CCS	Agreement against invasive FFR
						(FFR cut-off ≤0.80):
						r = 0.88, *p *< 0.0001
						BA mean difference: 0.007 ± 0.05
						AUC 0.93
		FAST-FFR Study [52]	Prospective, multicentric	2018	319 vessels with intermediate lesions from 301 patients with CCS	r = 0.80, *p *< 0.001
						BA mean difference: 0.01 ± 0.06
						AUC 0.94 [95% CI: 0.92–0.97]
${\mathrm{FFR}}_{\text{CT}}$, HeartFlow	CTCA-based 3D coronary tree reconstruction (including bifurcations). Coronary flow derived from ventricular mass. BCs as lumped parameter models of aortic inlet, and coronary microcirculation.	DISCOVER-FLOW [61]	Prospective, multicentric	2011	159 vessels with lesions ≥50%DS from 103 patients with CCS	Agreement against invasive FFR
						(FFR cut-off ≤0.80):
						r = 0.72, *p *< 0.001
						BA mean difference: 0.02 ± 0.12, *p* = 0.016
						AUC 0.90
		DeFACTO [62]	Prospective, multicentric	2013	82 vessels with 30–70%DS (intermediate stenosis) from 82 patients with CCS	Agreement against invasive FFR (FFR cut-off ≤0.80) in intermediate stenosis:
						r = 0.50, *p *< 0.001
						BA mean difference: –0.05 [–0.25 to 0.15]
						AUC 0.71 [95% CI: 0.58–0.83]
						NPV 0.91 [95% CI: 0.80–0.97]
		PLATFORM [63]	Prospective, multicentric	2016	584 patients with 20–80% likelihood of CAD and ≥30%DS at CTCA	Composite of death, myocardial infarction and unplanned revascularization at 1 year:
						${\mathrm{FFR}}_{\text{CT}}$-guided group: 1.04%
						Angiography-guided group: 1.07%
						Lower costs and same QoL in CTCA+${\mathrm{FFR}}_{\text{CT}}$ group in comparison with ICA
		ADVANCE [64, 65]	Prospective, multicentric	2020	4288 patients with CCS who underwent CTCA and have 1 year data available	Composite of death, myocardial infarction and ACS leading to urgent revascularization at 1 year:
						${\mathrm{FFR}}_{\text{CT}}$ ≤0.80 Relative risk: 1.81 [95% CI: 0.96–3.43], *p* = 0.06
		P3 Trial [66]	Prospective, multicentric	2022	123 vessels with FFR ≤0.80 from 120 patients with CCS	Agreement between post-PCI ${\mathrm{FFR}}_{\text{CT}}$ and post-PCI FFR:
						BA mean difference: 0.02 ± 0.07

**Abbreviations**: 3DQCA, Three-dimensional Quantitative Coronary Angiography; ADVANCE, Assessing Diagnostic Value of Non-invasive ${\mathrm{FFR}}_{\text{CT}}$ in Coronary Care; AUC, Area Under the Curve; BA, Bland-Altmann analysis; BC, boundary condition; CCS, Chronic Coronary Syndrome; CFD, Computational Fluid Dynamics; CAAS-vFFR, Cardiovascular Angiographic Analysis System for Vessel; CAD, Coronary Artery Disease; cQFR, Contrast Quantitative Flow Reserve; CTCA, Computed Tomography Coronary Angiography; DeFACTO, DEtermination of Fractional flow reserve by Anatomic Computed TOmographic Angiography; DISCOVER-FLOW, Diagnosis of Ischemia-Causing Stenoses Obtained Via Noninvasive Fractional Flow Reserve; DS, Diameter Stenosis; FAST, Fast Assessment of STenosis Severity; FAST-FFR, ${\mathrm{FFR}}_{a\,n\,g\,i\,o}$ Accuracy versus Standard FFR; FAVOR, Functional Assessment by Various Flow Reconstructions; FFR, Fractional Flow Reserve; ${\mathrm{FFR}}_{\text{CT}}$, Computed Tomography-derived Fractional Flow Reserve; HR, Hazard Ratio; ICA, Invasive Coronary Angiography; NPV, Negative Predictive Value; PLATFORM, Prospective LongitudinAl Trial of FFRct, Outcome and Resource IMpacts; QFR, Quantitative Flow Reserve; QoL, Quality of Life; RCT, Randomized Controlled Trial; vFFR, virtual Fractional Flow Reserve.

From the technical viewpoint, CAAS-vFFR uses angiography-based 3D vascular models without reconstruction of side branches [53]. The user is requested to provide the invasively measured aortic root pressure [53]. Next, to compute the CAAS-vFFR the pressure drop along the vessel segment of interest under hyperemic condition is instantaneously calculated by solving a simplified fluid dynamics equation accounting for pressure losses due to viscous friction of the blood flowing through the narrowed vessel and pressure losses due to flow separation downstream from the narrowing, with empirically determined coefficients [67].

Similarly, 3D side branch-free vessel reconstructions are employed by QFR for the vFFR computation [57]. The algorithm automatically divides the reconstructed vessel into equally spaced consecutive segments and estimate the pressure drop for each segment as a quadratic function of the hyperemic flow velocity, with coefficients dependent on the stenosis geometry. By assuming a fixed mean hyperemic coronary flow velocity of 0.35 m/s [68], the algorithm generates an initial output, called ‘fixed-QFR’ (fQFR). To improve patient-specificity, the software allows applying the TIMI frame counting analysis—as related to vessel flow velocity [69]—and to obtain the contrast-QFR (cQFR) at non-hyperemic conditions or the adenosine-QFR (aQFR) after intravenous administration of adenosine [68]. cQFR was shown to be superior to both fQFR and aQFR [57].

Differently from CAAS-vFFR and QFR, vessel geometrical reconstructions for FFRangio include bifurcations with side branches with diameter ≥0.5 mm [52]. The coronary tree is generated rapidly thanks to automatic vessel and lesion detection combined with correction feedback from the user. Based upon Poiseuille’s law, flow analysis is executed at each coronary segment and junction, and the overall resistance of the generated arterial network is determined. Hence, FFRangio values are inferred as the contribution of each narrowing to the total resistance [52].

### 5.2 Intracoronary Pressure Evaluation Based on Non-Invasive Imaging Modalities

**Fig. 4.**
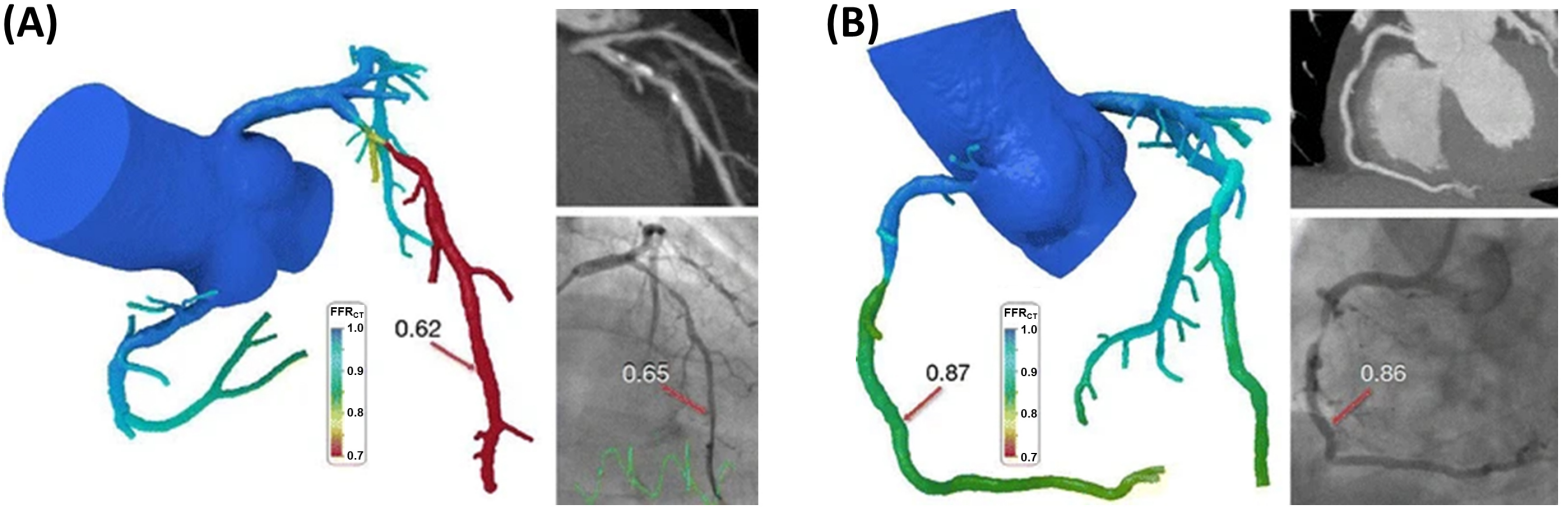
**Two case examples showing the results of the HeartFlow CFD based tool for the computation of the virtual fractional flow reserve from CTCA (i.e., the ${\mathrm{FFR}}_{\text{𝐂𝐓}}$)**. The examples highlight the benefit of ${\mathrm{FFR}}_{\text{CT}}$ in differentiating functional significance in coronary vessels with anatomically obstructive stenoses. (A) CCTA demonstrated significant coronary artery disease with stenosis >50% in the left anterior descending (LAD) artery. This was confirmed by quantitative angiography with a stenosis of 57%. The CFD model based on the CTCA revealed a hemodynamically significant lesion with ${\mathrm{FFR}}_{\text{CT}}$ in the distal LAD of 0.62. The measured FFR during invasive angiography was 0.65. (B) CCTA demonstrated a stenosis >50% in the mid right coronary artery (RCA). This was confirmed by quantitative angiography with a stenosis of 62%. Computed ${\mathrm{FFR}}_{\text{CT}}$ was 0.87, indicating a nonfunctionally significant stenosis. This was confirmed by a measured FFR of 0.86. Reprinted with permission from Zarins CK, Taylor CA, Min JK. Computed fractional flow reserve (${\mathrm{FFT}}_{\text{CT}}$) derived from coronary CT angiography. Journal of Cardiovascular Translational Research. 2013; 6(5): 708–714. doi: 10.1007/s12265-013-9498-4 [71]. (http://creativecommons.org/licenses/by/4.0/).

The application of CFD to non-invasive imaging modalities to predict blood flow and lesion-specific FFR preceded the development of angiography-derived FFR software (see Table 1). Taylor and colleagues [70] provided the first example of virtual fractional flow reserve derivation from CTCA, the so-called ${\mathrm{FFR}}_{\text{CT}}$ (HeartFlow, Mountain View, CA, USA). This tool has received both the CE mark and FDA approval, and it is currently commercially available in Japan. The software solution is based on volumetric CTCA data, morphometric laws and CFD analysis, as detailed in [70, 71]. In short, a patient-specific 3D model of the aortic root and coronary tree reconstructed from CTCA is coupled with lumped parameter models representing heart, systemic circulation, and coronary microcirculation. To define the flow-split between the coronary branches, firstly the total coronary flow under resting condition is derived from the myocardial volume, estimated from CTCA. Secondly, the total coronary resistance is computed considering the total coronary flow and the mean aortic pressure. Lastly, unique resistance values are prescribed to the lumped parameter models of coronary microcirculation downstream of the epicardial arteries relying on vessel diameter-based morphometric laws (e.g., Murray’s law [72], according to which the resistance to flow of a coronary branch is inversely related to the coronary artery diameter). To simulate hyperemic condition, the effect of adenosine on reducing the peripheral resistance of the coronary microcirculation is modelled by setting the total coronary resistance as 24% of the resting value [73] and assuming that the hyperemic microcirculatory resistance distal to a stenosis is the same as that of a healthy coronary artery [74]. The CFD simulation is performed centrally by the company and ${\mathrm{FFR}}_{\text{CT}}$ results are generated with a supercomputer within few hours (Fig. 4).

Clinical evidence proved higher per-vessel diagnostic performance for ${\mathrm{FFR}}_{\text{CT}}$ in direct comparison with coronary CTCA, single-photon emission computed tomography (SPECT), and positron emission tomography (PET) for ischemia diagnosis (AUC 0.94, 0.83, 0.70, 0.87, respectively; *p *< 0.01 in all cases) [75]. In a large multicentric real-world patient cohort, the implementation of ${\mathrm{FFR}}_{\text{CT}}$ led to modified treatment recommendation in two-thirds of subjects as compared to CCTA alone and was associated with less negative findings at the ICA [64]. Furthermore, at one year baseline ${\mathrm{FFR}}_{\text{CT}}$ below 0.80 showed a trend (*p* = 0.06) towards higher occurrence of adverse cardiovascular events [65]. In patients with intermediate pre-test probability for CAD, a ${\mathrm{FFR}}_{\text{CT}}$-guided care decision making resulted in lower financial costs, while holding similar clinical outcomes and quality of life indices [63]. Finally, a ${\mathrm{FFR}}_{\text{CT}}$-based PCI planner with simulation of (predicted) post-PCI FFR was recently clinically validated against invasive post-PCI FFR and showing high agreement level (mean difference: 0.02 ± 0.07 FFR unit) [66].

More recently, alternative solutions based on magnetic resonance angiography (MRA) have been also proposed. Contrast-enhanced ECG-gated 3T magnetic resonance scanners were used to produce 3D coronary images with a resolution of 0.64 × 0.64 × 0.75 ${\mathrm{mm}}^{3}$ [76]. Moreover, phase-contrast magnetic resonance imaging (PC-MRI) allowed coronary flow waveforms determination under rest and stress conditions [77], while self-gating principles improved vessel recognition by correcting for physiologic motion [78]. The obtained patient-specific coronary flow values were applied as inflow BCs to determine FFR based on CFD simulations [79]. This technology is currently undergoing further clinical investigation.

## 6. CFD Based Intracoronary Flow Patterns

Blood flow velocity relates to general physical laws governing balance among fluid forces. Ideally, undisturbed blood flow crossing a straight coronary segment presents the general characteristics of laminarity with co-axiality of the flow velocity vectors pointing to the same direction and decremental magnitude towards vessel walls where the blood interacts with the endothelial surface. In this condition, the blood velocity profile is axial-symmetric at each vessel cross-section. However, any deviations from straight vessel geometry markedly impact coronary flow patterns. In particular, the presence of curvature imparts a displacement of the location of the maximum velocity with respect to the vessel, in consequence of the vessel curvature-generated centrifugal force acting on the streaming blood. The deflection of the maximum peak velocity from the centerline (as in the classical Poiseuille flow) to the outer side of the curved vessel is the consequence of the balance between the centrifugal force, the viscous forces exchanged by the wall with blood and of the pressure gradient generating radially on the vessel cross-section, which leads to the establishment of the so-called secondary flows on the vessel cross-section. The composition of the two blood flow components, the one along the main flow direction (through-plane component) with the secondary flows (in-plane component) leads to the production of fully 3D blood flow patterns characterized by helical motion. The described phenomenon is exacerbated by the presence of bifurcations and side branches. As a result, flow disturbances are generated close to the internal and external vascular walls facing the carina [80], where blood flow separation and reattachment to the vessel wall, stagnation and recirculation may occur. Such flow disturbances are recognized as aggravating flow events related to the atherosclerotic disease onset/development [81, 82]. In other cases, vascular remodeling may occur, disrupting the smooth interface between blood flow and endothelium. Typically represented by coronary atherosclerotic plaques, these anatomical elements shape the local hemodynamics imparting multidirectionality and flow disturbances. Depending on the level of luminal protrusion, the local hemodynamics may be altered not only ‘near-wall’ but also in the bulk region of the vessel.

Differently from intracoronary pressure gradients, an invasive assessment of velocity vector fields and shear forces generated by the interaction between the viscous flowing blood and coronary arteries wall is at the moment elusive [23]. Personalized computational simulations have the potential to bridge this gap, providing a reliable quantification of the velocity field and shear forces after verification, validation, and uncertainty quantification of coronary models [83]. Currently, the application of CFD simulations for the characterization of flow patterns in human coronary arteries remains a subject of research. Commercial software solutions for clinical use are still not available.

In Sections 6.1 and 6.2, computationally derived biomechanical quantities describing the near-wall and intravascular flow patterns are discussed. Focus is centered on their role in understanding of atherosclerotic pathophysiology and their clinical application.

### 6.1 Near-Wall Flow Patterns

The interaction between the viscous blood and the vessel wall imparts at the blood-endothelium interface a state of stress, i.e., a force per unit surface. Analytically, the vector resultant of those frictional forces applied to a given endothelial unit area and with orientation tangential to the luminal surface is defined as wall shear stress (WSS), measured in N/$m^{2}$ or dyn/${\mathrm{cm}}^{2}$ or, most commonly, in Pascal (Pa; 1 Pa = 1 N/$m^{2}$ = 10 dyn/${\mathrm{cm}}^{2}$). Although several order of magnitude lower than the tensile forces exerted on vascular structures by the pulsatile blood (in kPa) [3], WSS has a valuable biological significance [23, 84, 85], triggering the endothelial mechanosensory machinery that regulates endothelial function and homeostasis [12, 86].

In regions of disturbed shear forces, such as near arterial bifurcations, the long-term exposure to low WSS values (typically <1 Pa) [23] has been associated to pro-inflammatory cellular cascade activation as well as enhanced lipidic and macrophage infiltration [87], hence leading to wall remodeling, fibrous cap thinning, and subintimal ischemia, which stimulates the local proliferation of the vasa vasorum, with risk of intraplaque hemorrhage [88]. Clinically, luminal areas exposed to low WSS have been associated with regional endothelial dysfunction [89] and plaque progression requiring revascularization (PREDICTION study) [6]. Moreover, low WSS has provided incremental risk stratification of untreated coronary lesions beyond measures of plaque burden, luminal surface area and plaque morphology (PROSPECT study) [32].

Conversely, high WSS magnitude values (typically >5 Pa) have been linked with plaque vulnerability and rupture [33, 90, 91]. Longitudinal and cross-sectional studies on human coronary arteries based on vessel-specific CFD simulations have reported an increase in plaque necrotic core, calcium, increased strain, development of expansive remodeling, and presence of intraplaque hemorrhage, large necrotic core, napkin-ring sign in areas exposed to high WSS [88], and incremental value for high WSS for predicting myocardial infarction over FFR alone [33, 91].

**Fig. 5.**
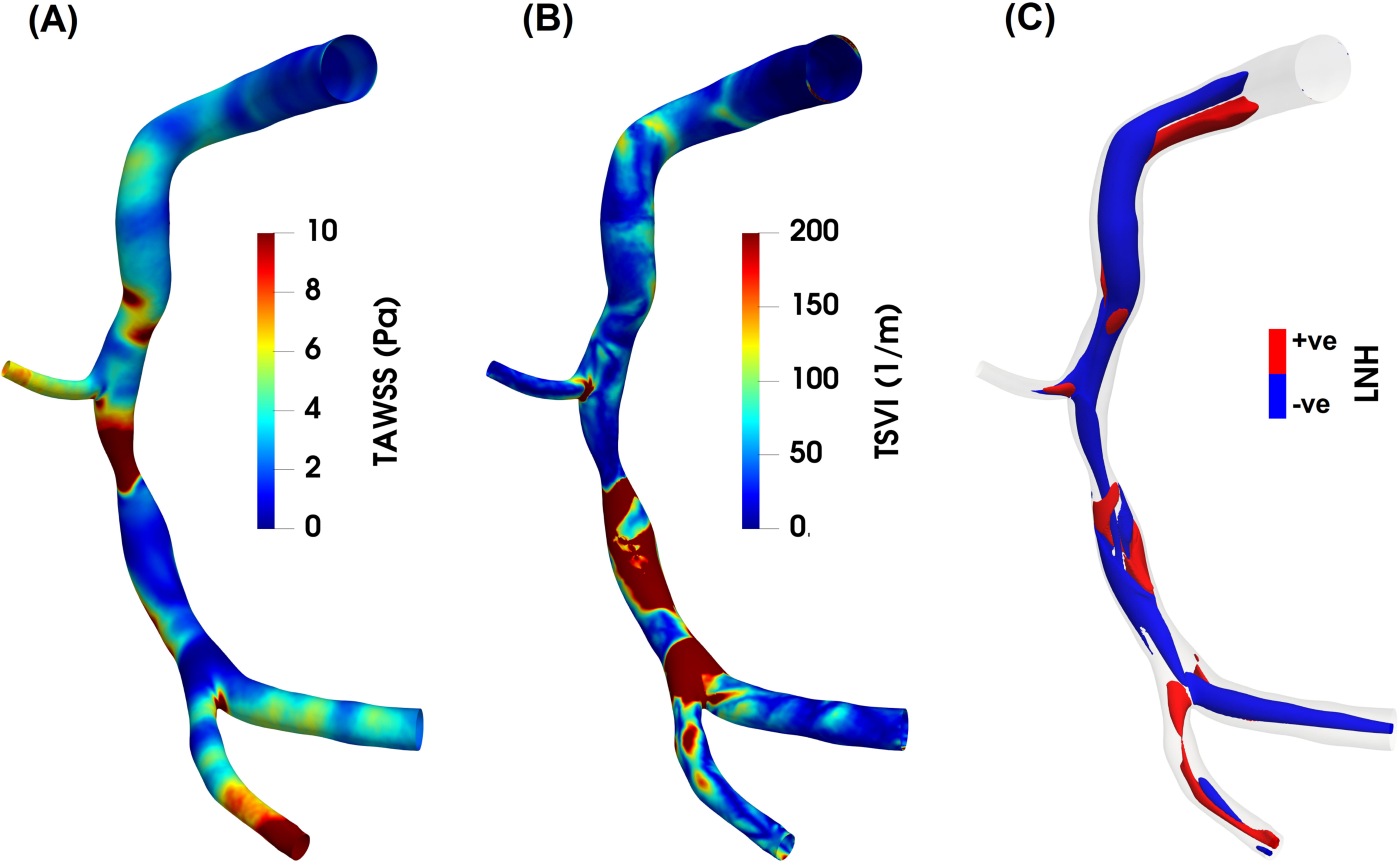
**Luminal maps of (A) time-average wall shear stress (TAWSS), (B) topological shear variability index (TSVI) and (C) cycle-average local normalized helicity (LNH) for an explanatory diseased right coronary artery model**. As expected, high TAWSS values characterize the stenotic region of the coronary artery, while low TAWSS are present downstream of the stenosis (panel A). As for the TSVI, a high variability in WSS contraction/expansion action at the endothelium during the cardiac cycle clearly emerges downstream of the stenosis, at the bifurcation region and at the side branch (panel B). Counter-rotating helical flow structures develop in the intravascular region of the coronary model here reported (panel C). Right-/left- handed helical blood patterns are identified by positive/negative LNH values and displayed in red/blue, respectively. The diseased right coronary artery belongs to a patient recruited during the RELATE clinical trial (ClinicalTrials.gov Identifier: NCT04048005).

**Fig. 6.**
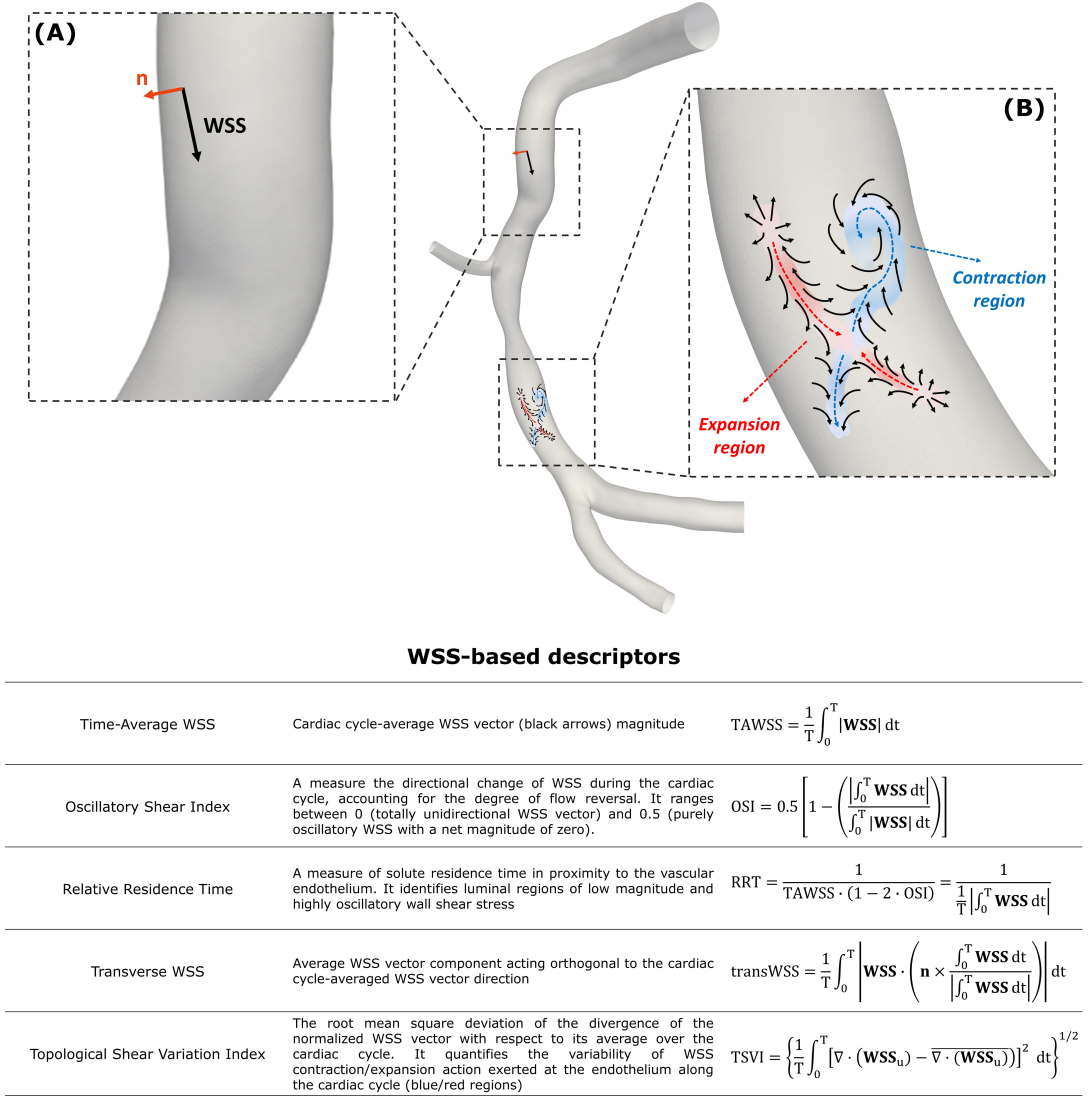
**Near-wall hemodynamic descriptors**. (A) Example of **WSS** vector acting on a generic point at the luminal surface (black arrow) of a diseased right coronary artery. At the same point, the unit vector **n** normal to the vessel wall is reported (orange arrow). (B) Explanatory maps of WSS vector field (black arrows) with identified contraction/action regions at the luminal surface of the same artery coloured by blue/red, respectively. The diseased right coronary artery belongs to a patient recruited during the RELATE clinical trial (ClinicalTrials.gov Identifier: NCT04048005). The table at the bottom reports the WSS-based descriptors of disturbed flow. For each descriptor, a short caption together with the mathematical formulation is reported. T is the cardiac cycle; **${\mathrm{WSS}}_{\text{𝐮}}$** is the normalized WSS vector field.

Notably, areas of low and high WSS may be contiguous. Low WSS surface areas are typically located at inner curvatures, at the waist of bifurcations or downstream of a stenosis (Fig. 5A). Conversely, high WSS surface areas are located at outer curvatures, at the flow divider of bifurcations, upstream or at the lesion throat (Fig. 5A) [6, 12]. For this reason, the interpretation of WSS values in absolute terms only could be misleading, and its contextualization in a proper physiological context is mandatory. As a consequence, in addition to the traditional time-average WSS (TAWSS, namely the WSS magnitude averaged along the cardiac cycle) [23], several WSS-based quantities have been introduced/tested, aiming at quantifying different features of the WSS profile, with particular attention to its multidirectionality and magnitude variability along the cardiac cycle (Fig. 6) [92, 93, 94]. For instance, WSS-based quantities were proposed describing (i) the degree of flow reversal (oscillatory shear index, OSI) [95], (ii) the near-wall solute residence time (relative residence time, RRT) [96], (iii) the multidirectional character of the disturbed blood flow through the quantification of the cycle-averaged WSS component orthogonal to the mean WSS vector direction (transverse WSS, transWSS) [97], or (iv) the variability of contraction/expansion action of endothelial shear forces along the cardiac cycle (topological shear variation index, TSVI) [98] (Fig. 5B). High OSI (≥0.15) was associated with a vulnerable plaque phenotype with lipid accumulation and inflammatory cell infiltration [99]. A positive relation emerged for RRT and atherosclerotic plaque calcification and necrosis [100]. TransWSS was related to changes of plaque composition over time in human coronary arteries [100]. Finally, high TSVI (>40.5 $m^{-1}$) identified mild coronary lesions future site of myocardial infarction within 5 years [90]. Mechanistically, this may be linked to the altered shrinkage and widening of intercellular gaps in case of amplified contraction/expansion action of the endothelium [101], as well as to higher fibrous cap fragility, accelerated disease progression, and plaque rupture [102].

### 6.2 Intravascular Flow Patterns

Besides the role of WSS, distinguishable intravascular flow features have also been suggested to markedly impact the atherosclerotic disease natural history. Previous studies have clearly revealed that (i) arterial blood flow, under physiological conditions, is helical and (ii) the associated helicity intensity is instrumental in suppressing arterial flow disturbances in ostensibly healthy arteries, being thereby potentially protective for atherosclerotic lesions at the early stage [103, 104, 105, 106, 107, 108, 109].

**Fig. 7.**
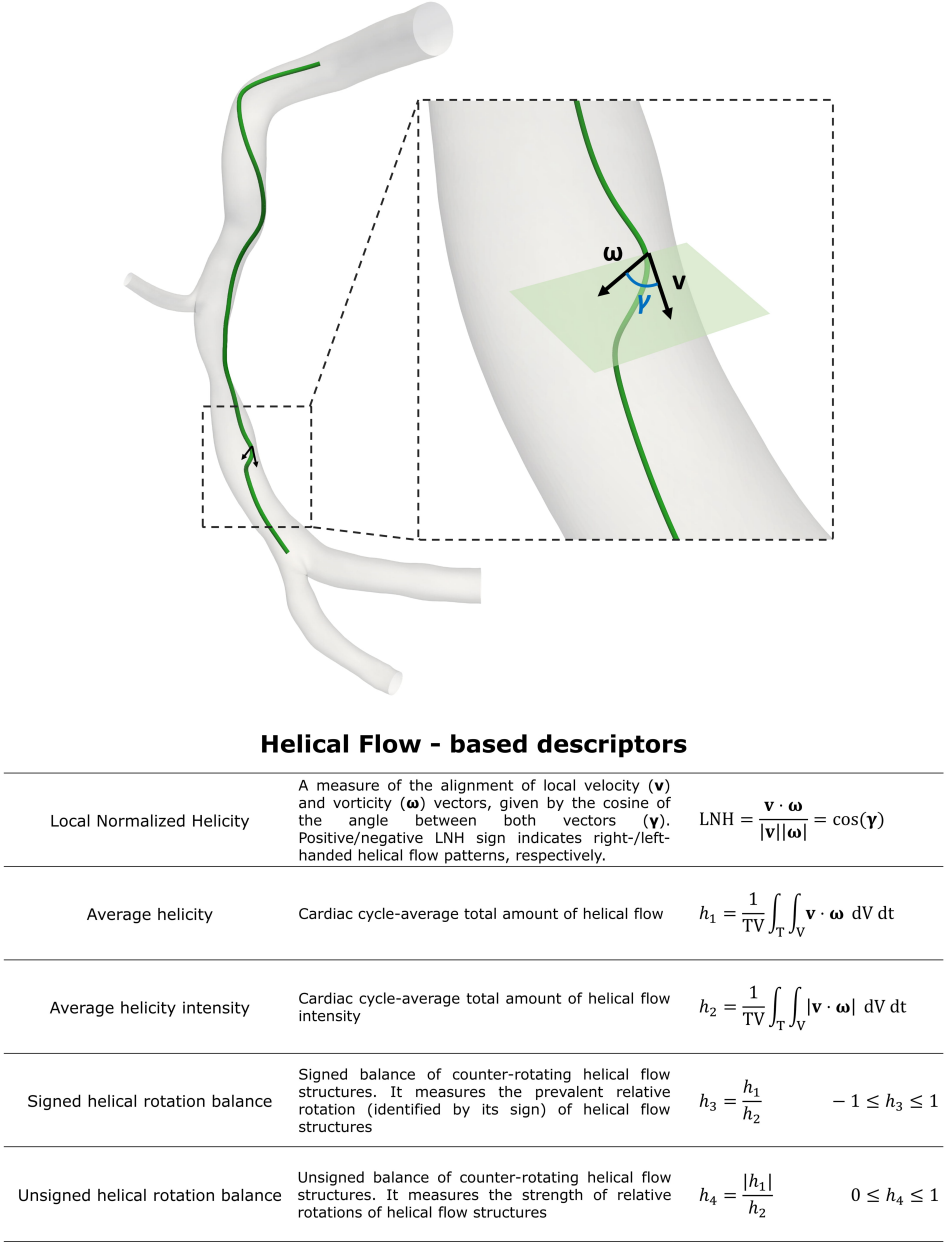
**Intravascular hemodynamic descriptors**. Figure: example of the helical-shaped trajectory described by an element of blood moving within an explanatory model of right coronary artery. This diseased artery belongs to a patient recruited during the RELATE clinical trial (ClinicalTrials.gov Identifier: NCT04048005). γ is the angle between local velocity (**v**) and vorticity (𝝎) vectors (black arrows). The table at the bottom reports the helical flow-based descriptors commonly used to characterize intracoronary hemodynamics. For each descriptor, a short caption together with the mathematical formulation is reported. T is the cardiac cycle; V is the whole arterial volume.

The analysis of arterial helical flow patterns can be provided by using the local normalized helicity (LNH) [108]. This hemodynamic quantity, defined as the cosine of the angle between the local velocity and vorticity vectors, allows for the identification of the rotating direction of helical fluid structures based on its sign (i.e., positive—right-handed; negative—left-handed) (Figs. 5C,7). Recent evidence, based on the visualization of intravascular LNH iso-surfaces, has pointed out that helical flow is a feature characterizing the physiological intravascular hemodynamics of healthy coronary arteries [103, 110]. The topology of coronary helical flow structures strongly depends on the vessel geometry (i.e., curvature, torsion, bifurcations, presence of stenosis), which may affect their generation, transport, and intensity along the arterial length [111].

A quantitative characterization of helical flow in terms of strength, size and relative rotational direction can be obtained by several helicity-based descriptors, named as *h* indices (Fig. 7) [92, 93, 94, 105]. In detail, cycle averaged helicity (*$h_{1}$*) and helicity intensity *($h_{2}$*) quantify the net amount and the intensity of helical flow, respectively, while the signed (*$h_{3}$*) and unsigned (*$h_{4}$*) helical rotation balance measure the prevalence (by the sign of *$h_{3}$*) or only the strength of relative rotations of helical flow structures, respectively. In particular, among the helicity-based descriptors, *$h_{2}$* emerged as instrumental in stabilizing blood flow in coronary arteries imparting low WSS multidirectionality and minimizing the endothelial surface exposed to low atherogenic WSS [103]. More specifically, a non-linear decreasing trend relating *$h_{2}$* with the coronary luminal surface exposed to low WSS, was found, indicating that the higher is the helicity intensity, the lower is the coronary endothelial region facing proatherogenic WSS [103]. As confirmation, recent findings revealed the existence of a clear association between helical flow intensity and coronary atherosclerotic plaque initiation and growth [104]. The latter (i) confirmed the role of helical blood flow features in conditioning WSS luminal distribution, which in turn interacts with the pathophysiology of atherosclerotic plaque formation, and (ii) suggested that helical flow intensity is protective against coronary atherosclerotic plaque onset/progression, and may serve as a biomechanical predictor of it [104]. The evidences of the physiological significance of helical blood flow, already emerged from CFD studies on swine coronary arteries [103, 104], are expected to be directly translated to human coronary disease, due to the demonstrated applicability of swine-specific computational models to investigate the hemodynamic-related risk of coronary atherosclerosis in humans [110].

All these aspects together with the clinical feasibility of helical pattern quantification—at least in large arteries—by means of four-dimensional (4D) flow PC-MRI have stimulated the interest on the use of helical flow as a potential surrogate marker for the atherosclerotic risk at the early stage. The *in vivo* measurements of intravascular fluid quantities such as helical flow, which are less sensitive to noise, lumen edge definition, spatial and temporal resolution than *in vivo* WSS assessment [112, 113], could be a novel surrogate determinant of plaque vulnerability. In the near future advances in clinical imaging (e.g., applying 4D flow PC-MRI sequences properly developed to measure coronary blood flow) [114] and online CFD analysis are indeed expected to allow non-invasive *in vivo*-based prediction of coronary atherosclerotic or plaque rupture risk based upon helicity-based descriptors [12, 115].

## 7. Limitations of Current CFD Simulations and Future Perspectives

Despite recent developments, intracoronary computational hemodynamics simulations still present several criticalities hampering their clinical usability.

Firstly, considering that ‘vessel geometry shapes the flow’ [12], a reliable personalized CFD simulation requires accurate 3D reconstruction of the coronary artery lumen. Hence, inaccuracy in the vascular tracing, inadequate space resolution or blooming artifacts (especially for CTCA-based modalities) might affect the reconstructed vascular geometry [116]. This is even more critical in case of bifurcations, where daughter vessels lie on different spatial planes and geometrical reconstructions based on two ICA projections could be therefore inaccurate [117]. Moreover, additional manual corrections are often required for the contouring of the polygon of confluence of coronary bifurcations [118]. Ideally, an accurate 3D vessel reconstruction could be achieved using intravascular imaging techniques, such as IVUS or OCT. However, invasiveness, the limitation to measure one vessel at a time, and costs advocated the exploration of alternative imaging modalities, namely CCTA and ICA, to perform CFD simulations for clinical applications. While adopted for intracoronary pressure gradient evaluation, the use of CCTA in CFD modelling for the characterization of flow patterns and shear forces is limited because of the low image resolution and presence of artifacts. Nevertheless, the potential utility of CCTA-derived CFD for the identification of high-risk plaques was successfully validated in the EMERALD study [119]. Recently, angiography-based CFD simulations were also applied in human coronary arteries with promising results for the quantification of the WSS patterns [90, 91]. Validation against IVUS and OCT, used as ground-truth, is in process [116, 120].

Secondly, the definition of BCs, which highly impact the final results of the CFD simulations, is challenging and presents a high degree of uncertainty because intracoronary flow measurements are seldom executed in the clinical routine and often characterized by low accuracy and repeatability, thus requiring the use of theoretical assumptions and/or idealizations [121, 122, 123, 124]. When clinical measurements are available, a patient-specific approach to define inlet/outlet BCs should be preferred to generalized/estimated ones, which might result in not realistic profiling of flow disturbances, especially near side branches and curvatures where atherosclerotic plaques preferentially develop [123, 124]. Furthermore, the heterogeneity in their definition can often preclude comparison of the results from different studies.

Thirdly, computational time needed to execute CFD simulations varies according to model complexity, spatiotemporal discretization, tracing length and computer characteristics, precluding in most cases the ‘on-line’ execution of CFD simulation within the time window of a diagnostic coronary angiogram. Of note, the computational time adds up to the time needed to upload the imaging data and to reconstruct the 3D coronary artery model (e.g., in case of angiographic data, to upload two angiographic projections, to complete the vessel tracing and to obtain the 3D vessel model). Therefore, a higher level of automation is needed to move CFD simulations from the lab to clinical practice. Next generation CFD software are expected to produce reliable coronary hemodynamics simulations within few minutes (or even instantaneously) and with minimal operator interference. In this context, a recent study has shown the clinical use of a prototype commercial software (CAAS Workstation, WSS tool, Pie Medical, Maastricht, The Netherlands) able to provide transient hemodynamic results for mild coronary artery lesions in terms of WSS-based descriptors using angiographic data and CFD modelling in less than 15 minutes [90]. Furthermore, ad hoc programmed artificial intelligence and in particular machine learning algorithms can be trained to predict flow components directly from the coronary imaging and vessel geometry [125, 126, 127], hence bypassing the time-demanding computation of instantaneous intracoronary flow and pressure. This task can be achieved adopting several different strategies: among them we mention the use of physics-informed neural networks that, integrating mathematical equations governing blood flow with very few patient-specific measurement points within a flexible deep learning framework, have already demonstrated to improve WSS quantification in diseased arterial flows [128]. Moreover, cloud CFD application may diminish computational time by allowing remote use of high-performance computing clusters, and, if associated with a centralized core laboratory, could favor the quality of the analysis while reducing inter-operator variability. On one hand, all this will facilitate clinical application of CFD simulations. On the other hand, it will push the boundaries of intracoronary biomechanics simulations even further. In fact, recent modelling strategies are combining plaque structural stress and strain with hemodynamic shear stress, thus providing a more comprehensive analysis of the local biomechanics exerted on plaques or vascular components, essential in understanding plaque vulnerability and in predicting results after coronary interventions [129].

Lastly, in order to justify the routine clinical application of CFD simulations in the catheterization laboratories, more robust clinical evidence for CFD results is advocated. To this aim, the execution of randomized trials is required to confirm the relationship between CFD results (with particular reference to the near-wall and intravascular hemodynamic quantities) and clinical outcomes, and ultimately to define the role of CFD simulations in clinical practice. Additional technologies, such as augmented reality and more immersive user interface, might also play a role towards the clinical use of these modalities, offering a more intuitive reading of intracoronary flow specifics to physicians [130].

## 8. Conclusions

CFD models of coronary hemodynamics allow a far-deeper understanding of the critical relationship between intracoronary flow, vascular anatomy and plaque composition. In fact, the interplay between biology (patient risk profile, genetics and congenital vascular anatomy), intravascular pressure gradients and specific flow patterns has proven effects on atherogenesis, plaque composition and destabilization, as outlined in the ‘hemodynamic risk hypothesis’ [3, 12]. This gained basic knowledge has stimulated CFD based clinical applications, providing physicians with reliable non-invasive tools for intracoronary pressure gradients estimation as well as the quantitative assessment of intracoronary shear forces on the endothelium and their link with functional plaques characterization and vulnerability assessment, to be used for predictive purposes. Moreover, CFD application might entail also procedural planning (e.g., post-PCI ${\mathrm{FFR}}_{\text{CT}}$) and stent scaffolds design.

Overcoming current technical challenges with modern technologies will allow for quicker and more reliable computational solutions, which, validated in the proper clinical settings, will ultimately favor a wider use of a physiology-based lesion evaluation in the clinical practice, with expected benefit for patients and financial gain.
